# Xp11.2 translocation renal cell carcinoma occurring during pregnancy with a novel translocation involving chromosome 19: a case report with review of the literature

**DOI:** 10.1186/1746-1596-4-15

**Published:** 2009-05-18

**Authors:** Henry B Armah, Anil V Parwani, Urvashi Surti, Sheldon I Bastacky

**Affiliations:** 1 Department of Pathology, Presbyterian-Shadyside Hospital, University of Pittsburgh Medical Center, Pittsburgh, Pennsylvania, USA; 2 Pittsburgh Cytogenetics Laboratory, Magee-Womens Hospital, University of Pittsburgh Medical Center, Pittsburgh, Pennsylvania, USA

## Abstract

The recently recognized renal cell carcinomas (RCCs) associated with Xp11.2 translocations (TFE3 transcription factor gene fusions) are rare tumors predominantly reported in children. They comprise at least one-third of pediatric RCCs and only few adult cases have been reported. Here, we present a case of Xp11.2 translocation RCC in 26-year-old pregnant female. Her routine antenatal ultrasonography accidentally found a complex cystic right renal mass. Further radiologic studies revealed unilocular cyst with multiple mural nodules at inferior pole of right kidney, which was suspicious for RCC. She underwent right radical nephrectomy at 15 weeks gestation. Macroscopically, the cystic tumor was well encapsulated with multiple friable mural nodules on its inner surface. Microscopically, the tumor consisted of clear and eosinophilic/oncocytic voluminous cells arranged in papillary, trabecular, and nested/alveolar patterns. Occasional hyaline nodules and numerous psammoma bodies were present.

Immunohistochemically, the tumor showed strong nuclear positivity for TFE3. Epithelial membrane antigen, CD10, and E-cadherin were strongly positive. Cytokeratin AE1/AE3, cytokeratin CAM-5.2, calveolin, and parvalbumin were moderately positive. Cytokeratin 7, renal cell carcinoma antigen, and colloidal iron were focally weakly positive. BerEP4 and carbonic anhydrase IX were negative. Cytogenetically, the tumor harbored a novel variant translocation involving chromosomes X and 19, t(X;19)(p11.2;q13.1). Interphase FISH analysis performed on cultured and uncultured tumor cells using a dual-color break-apart DNA probe within the *BCL3 *gene on 19q13.3 was negative for the *BCL3 *gene rearrangement. She received no adjuvant therapy, delivered a normal term baby five months later, and is alive without evidence of disease 27 months after diagnosis and surgery. Unlike most recently reported Xp11.2 translocation RCCs in adult patients with aggressive clinical course, this adult case occurring during pregnancy with a novel translocation involving chromosome 19 followed an indolent clinical course.

## Background

Xp11.2/TFE3 translocation renal cell carcinomas (RCCs), a recently classified distinct subtype, are rare tumors that usually affect children and adolescents [1-8], with only few reported adult cases to date [9-24]. It is estimated that approximately one-third of pediatric RCCs are Xp11.2 translocation RCCs [2-5], whereas conventional clear cell RCCs make up about 15% of pediatric RCCs [25,26]. In contrast, conventional clear cell RCCs make up 70% of RCCs in adults and 53% in young adults [26], but the incidence of Xp11.2 translocation RCCs in adults and young adults is much smaller (estimated to be perhaps 1%) [1,5,7,17,19,27]. Xp11.2 translocation RCCs are defined by at least six different translocations involving the Xp11.2 chromosome, all of which result from gene fusions involving the TFE3 transcription factor gene, and their morphology and biological behavior are not widely recognized as yet [1-24]. They typically have papillary and/or nested architecture, and are composed of cells with clear and/or eosinophilic voluminous cytoplasm [1-24]. Translocations involving TFE3 induce overexpression of this protein, and hence nuclear immunolabelling for TFE3 is a sensitive and specific marker of neoplasms with TFE3 gene fusions [1-8]. Although only limited data are available thus far, they are believed to be rather indolent even when diagnosed at advanced stages [1-8], but there have been increasing recent reports of an aggressive clinical course in adult cases [9-24].

## Case presentation

**Figure 1:**
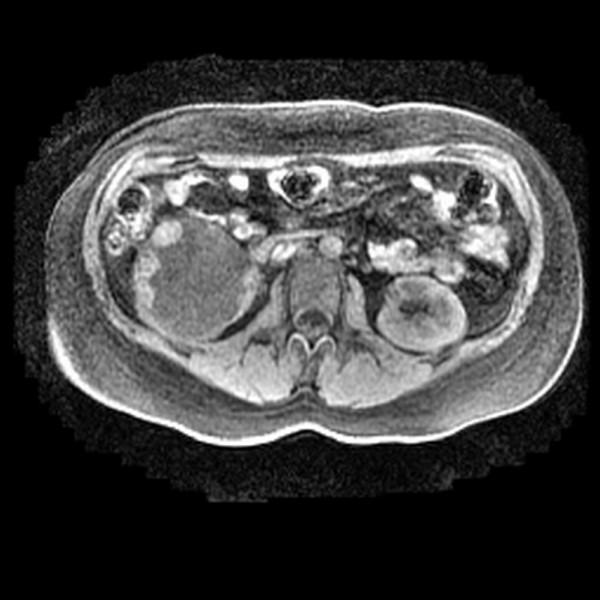
**MRI scan showing unilocular cystic right renal mass with multiple mural nodule**.

The patient was a 26-year-old pregnant woman (14 weeks gestation) who was accidentally found to have a complex cystic renal mass during routine antenatal ultrasonography. Subsequent magnetic resonance imaging (MRI) scan revealed a unilocular cystic mass with multiple mural nodules on its inner surface at the inferior pole of the right kidney, which was interpreted as radiologically suspicious for a RCC (Figure 1). She had no previous history of chemotherapy. All blood test were unremarkable. The patient underwent right radical nephrectomy at 15 weeks gestation. Surgery revealed the tumor to be confined within the kidney. The postoperative period was uneventful and the patient was discharged four days after surgery. She received no adjuvant therapy, and is alive without evidence of disease 27 months after diagnosis and radical nephrectomy. She delivered a normal term baby five months after diagnosis and radical nephrectomy, and macroscopic and microscopic evaluation of the placenta was unremarkable with no evidence of metastatic RCC.

## Methods

### Histologic examination

Tissues were fixed in 10% buffered formalin solution and embedded in paraffin blocks. Four-micrometer-thick sections were obtained and stained with hematoxylin and eosin for microscopic examination.

### Immunohistochemical analysis

Additional sections were used to perform immunohistochemical studies using an avidin-biotin peroxidase technique with hematoxylin counterstain. The antibodies used in this study include the following: cytokeratin AE1-AE3 (clone AE1-AE3/PCK26, Ventana Medical Systems Inc, Tucson, Arizona, USA), cytokeratin CAM-5.2 (clone CAM-5.2, Becton-Dickinson Biosciences, San Jose, California, USA), cytokeratin 7 (clone OV-TL 12/30, Ventana Medical Systems Inc, Tucson, Arizona, USA), epithelial membrane antigen (EMA, Clone E29, Ventana Medical Systems Inc, Tucson, Arizona, USA), renal cell carcinoma antigen (RCC, clone PN-15, Ventana Medical Systems Inc, Tucson, Arizona, USA), CD 10 (clone 56C6, Ventana Medical Systems Inc, Tucson, Arizona, USA), E-cadherin (clone ECH-6, Ventana Medical Systems Inc, Tucson, Arizona, USA), calveolin-1 (clone E249, Epitomics Inc, Burlingame, California, USA), parvalbumin (PARV-19, Sigma-Aldrich Company, St Louis, Missouri, USA), TFE3 (clone H-300, Santa Cruz Biotechnology Inc, Santa Cruz, California, USA), carbonic anhydrase IX (CA9, clone M75, DakoCytomation, Carpinteria, California, USA), and BerEP4 (clone M0804, DakoCytomation, Carpinteria, California, USA). Mowry's colloidal iron stain was also performed on tissue sections.

We analyzed the intensity and immunoreactivity of the immunostained sections. The intensity was graded qualitatively as weak, moderate, or strong on the basis of the brown color produced by the 3, 3'-diaminobenzidine chromogen. Diffuse and intense brown staining of the cytoplasmic or nuclear surfaces, as appropriate for each stain, was interpreted as strong staining intensity. Moderate intensity staining was characterized as non-diffuse but intense staining pattern, whereas weak intensity had a diffuse but non-intense staining pattern. Immunoreactivity was quantitatively estimated by the percentage of positive cells per representative section. Immunoreactivity was graded as 1+, 2+, and 3+, corresponding to less than 25%, between 25% and 50%, and greater than 50% of neoplastic cells showing positive staining per representative section, respectively.

### Cytogenetic analysis

A fresh unfixed tumor sample was submitted in RPMI tissue culture medium to the Pittsburgh Cytogenetics Laboratory of the Department of Pathology of the University of Pittsburgh Medical Center for cytogenetic analysis. The tumor tissue was treated with collagenase to enzymatically dissociate cells, which were then cultured for 22 days. Metaphase cells were harvested from monolayer cell cultures and chromosomes were GTG-banded using standard procedures.

### Fluorescence In Situ Hybridization (FISH) analysis

Two separate Fluorescence in situ hybridization (FISH) assays were performed using interphase cells derived from interphase cells harvested from monolayer cell cultures prepared as described above and a formalin-fixed paraffin-embedded section. Formalin-fixed paraffin-embedded sections were mounted and serially sectioned at 4-mm intervals. Separate sections stained with H & E were used to determine the area of the tissue to be targeted for analysis. The formalin-fixed paraffin-embedded slides were de-paraffinized in xylene twice for 10 minutes, dehydrated twice with 100% ethanol, and then pretreated using the DakoCytomation Paraffin Pretreatment Kit (DakoCytomation, Carpinteria, California, USA). Slides were digested for 18 minutes in protease solution (0.5 mg/ml) at 37°C.

For both assays, FISH was performed using the BCL3 (19q13.3) dual-color break-apart DNA probe (DakoCytomation, Carpinteria, California, USA). The target slide and probe were co-denatured at 95°C for 8 minutes and incubated overnight at 37°C in a humidified chamber. Post-hybridization washes were performed using 2× standard saline citrate/0.3% Igepal (Sigma) at 72°C for 2 minutes. Slides were air-dried in the dark and counterstained with 4',6'-diamidino-2-phenylindole (DakoCytomation, Carpinteria, California, USA). Analysis was performed using a Nikon Optiphot-2 microscope (Nikon, Inc.) and Quips Genetic Workstation equipped with a Chroma Technology filter fitted with single- band excitors for SpectrumOrange, fluorescein isothiocyanate, and 4',6'-diamidino-2-phenylindole (uv 360 nm; DakoCytomation, Carpinteria, California, USA). Only individual and well-delineated cells were scored. Overlapping cells were excluded from the analysis. A total of 222 and 162 cells were analyzed from the assays derived from monolayer cell culture and formalin-fixed paraffin-embedded section, respectively.

## Results

### Macroscopic findings

**Figure 2:**
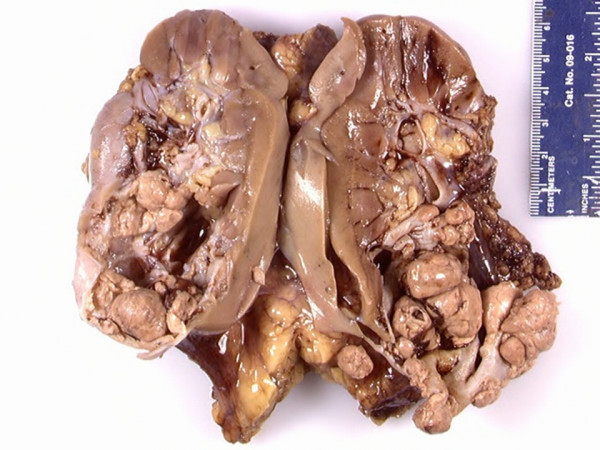
**Gross photograph of tumor after fixation showing unilocular cystic tumor with multiple friable mural nodules**.

The right nephrectomy specimen was bivalved from the peripheral cortex towards the hilum to show a 5.5 × 4.5 × 3.5-cm well encapsulated, unilocular cystic tumor at the inferior pole of the kidney. Serial sections of the tumor revealed multiple soft and friable tan-gray mural nodules arising or attached to the inner surface of the cyst, with these nodules measuring up to 2.0 cm in greatest dimension (Figure 2). The tumor was confined to the kidney and was present immediately deep to renal capsule. There was no gross evidence of extension of the tumor into the renal artery, renal vein, or renal sinus. No other lesions were present in the adjacent renal parenchyma.

### Microscopic findings

**Figure 3:**
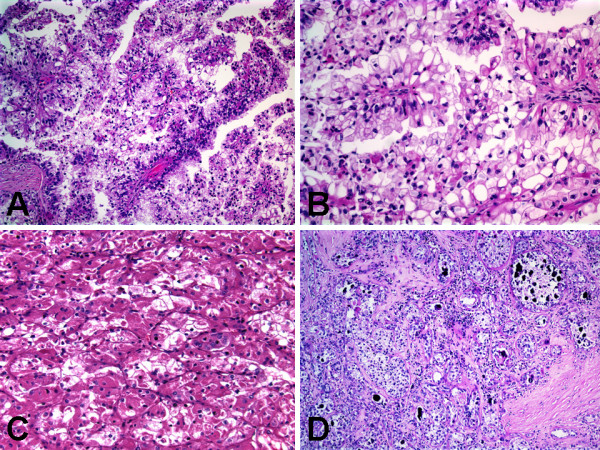
**Histologic features of Xp11 translocation RCC with novel breakpoints, t(X;19)(p11.2;q13.1)**. A, Papillary pattern with admixed voluminous clear and eosinophilic cells. B, Papillary pattern with voluminous clear cells. C, Nested/alveolar pattern with voluminous eosinophilic/oncocytic cells. D, Occasional hyaline nodules and numerous psammoma bodies.

The renal tumor showed carcinomatous proliferation of malignant epithelial cells arranged in papillary (Figures 3A &3B), trabecular, and nested/alveolar (Figure 3C) patterns. The tumor cells were large with discrete cell borders and had either abundant granular eosinophilic cytoplasm (imparting an oncocytic appearance, Figures 3A &3C) or abundant granular finely vacuolated cytoplasm (imparting a clear cell appearance, Figures 3A &3B). The nuclei were mildly enlarged with mild contour irregularities, and had fine chromatin with occasional prominent nucleoli (Fuhrman nuclear grade 2 out of 4). The stroma was vascular with occasional hyaline nodules and numerous psammoma bodies (Figure 3D). Mitotic figures were not identified. Abundant areas of central hemorrhagic necrosis containing hemosiderin-laden macrophages were present. There was no evidence of lymphovascular invasion, and all surgical resection margins were free of tumor.

### Immunohistochemical findings

**Figure 4:**
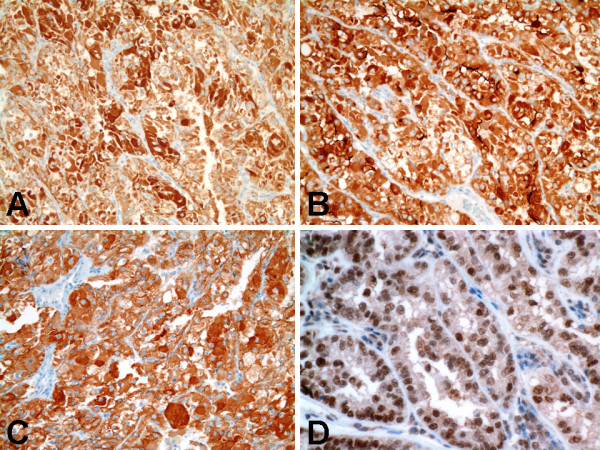
**Immunohistochemical findings of Xp11 translocation RCC with novel breakpoints, t(X;19)(p11.2;q13.1)**. A, Strongly positive cytoplasmic staining for epithelial membrane antigen. B, Strongly positive cytoplasmic staining for CD10. C, Strongly positive cytoplasmic staining for E-cadherin. D, Strong nuclear labeling for TFE3 protein.

**Figure 5:**
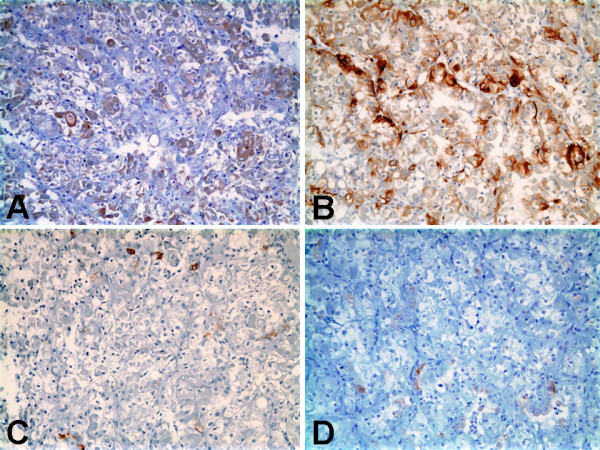
**Immunohistochemical findings of Xp11 translocation RCC with novel breakpoints, t(X;19)(p11.2;q13.1)**. A, Moderate positive cytoplasmic staining for cytokeratin AE1-AE3. B, Moderate positive cytoplasmic staining for cytokeratin CAM-5.2. C, Focal weak positive cytoplasmic staining for cytokeratin 7. D, Focal weak positive cytoplasmic staining for renal cell carcinoma antigen.

The immunohistochemical results are summarized in Table 1. The neoplastic cells were diffusely strongly immunoreactive (3+, cytoplasmic staining) for epithelial membrane antigen (EMA) [Figure 4A], CD10 (Figure 4B), and E-cadherin (Figure 4C). Nuclear staining for TFE3 (Figure 4D) was diffusely strongly immunoreactive (3+), indicating the presence of the corresponding Xp11.2 translocation. The neoplastic cells were focally moderately immunoreactive (2+, cytoplasmic staining) for cytokeratin AE1-AE3 (Figure 5A), cytokeratin CAM-5.2 (Figure 5B), calveolin, and parvalbumin. The neoplastic cells were focally weakly immunoreactive (1+, cytoplasmic staining) cytokeratin 7 (Figure 5C), renal cell carcinoma antigen (Figure 5D), and colloidal iron. The neoplastic cells did not express BerEP4 or carbonic anhydrase IX.

**Table 1:** Immunohistochemical findings of case of Xp11.2 translocation renal cell carcinoma with a novel translocation involving chromosome 19

Antibody to	Result (Positive, Negative)	Intensity (Strong, Moderate, Weak)	Immunoreactivity (1+, 2+, 3+)	Staining Pattern (Nuclear, Cytoplasmic)
TFE3	Positive	Strong	3+	Nuclear

Cytokeratin AE1-AE3	Positive	Moderate	2+	Cytoplasmic

Cytokeratin CAM-5.2	Positive	Moderate	2+	Cytoplasmic

Cytokeratin 7	Positive	Weak	1+	Cytoplasmic

Epithelial membrane antigen	Positive	Strong	3+	Cytoplasmic

Renal cell carcinoma antigen	Positive	Weak	1+	Cytoplasmic

CD 10	Positive	Strong	3+	Cytoplasmic

E-cadherin	Positive	Strong	3+	Cytoplasmic

Calveolin	Positive	Moderate	2+	Cytoplasmic

Parvalbumin	Positive	Moderate	2+	Cytoplasmic

Colloidal iron	Positive	Weak	1+	Cytoplasmic

BerEP4	Negative	Not Applicable	Not Applicable	Not Applicable

Carbonic anhydrase IX	Negative	Not Applicable	Not Applicable	Not Applicable

1+, < 25% Positive Cells; 2+, 25–50% Positive Cells; 3+, > 50% Positive Cells

### Cytogenetic findings

**Figure 6:**
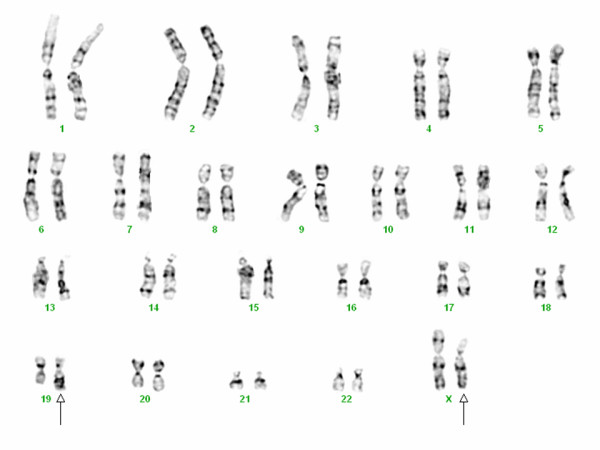
**Complete karyotype of Xp11 translocation RCC with novel breakpoints, t(X;19)(p11.2;q13.1)**.

Fifteen trypsin-Giemsa banded metaphase cells were analyzed from five and six day harvests of monolayer primary cell cultures and from a 22 day harvest of subcultured monolayer cell cultures derived from the renal mass. Two cells had an apparently normal female chromosome complement, most likely representing the stromal cells. One cell had a hypodiploid karyotype, likely as a result of random chromosomal loss. Nine cells had a 46, X, t(X;19)(p11.2;q13.1) chromosome pattern (Figure 6). Three additional cells, each with this same abnormality, had less than 46 chromosomes, likely as a result of random chromosomal loss. In addition to the above cells, a few cells with tetraploidy and endoreduplication with this translocation were observed. Therefore, this renal tumor showed a mosaic abnormal female chromosome analysis with an apparently normal cell line and an abnormal clone with a translocation between the short arm of an X chromosome and the long arm of chromosome 19. The overall karyotype results can be described by the following International System for Human Cytogenetic Nomenclature (ISCN 2005): 46, X, t(X;19)(p11.2;q13.1)[12]/46, XX[3]. This classical cytogenetic profile is consistent with a diagnosis of Xp11.2 translocation RCC with a novel translocation involving chromosome 19.

### Fluorescence In Situ Hybridization (FISH) findings

In order to determine if the *BCL3 *(19q13.3) gene located on the chromosome 19 was involved in the novel translocation observed by the classical cytogentic analysis above [t(X;19)(p11.2;q13.1)], cultured and uncultured tumor cells were evaluated by interphase FISH analysis using a dual-color break-apart DNA probe within the *BCL3 *gene on 19q13.3. The FISH analysis was negative for the *BCL3 *gene rearrangement in 222 (100%) of the 222 interphase cells examined from the assays derived from monolayer cell culture. Twenty-two (10%) of the 222 interphase cells examined from the assays derived from monolayer cell culture had 1–2 extra BCL3 signals. The significance of the presence of 10% of cells with 1–2 extra BCL3 signals is unclear. It may suggest the presence of a small clone with triploidy or tetraploidy. Tetraploidy was observed in a few cells by the classical cytogentic analysis above. Additionally, the FISH analysis was negative for the *BCL3 *gene rearrangement in 161 (99.4%) of the 162 interphase cells examined from the assays derived from formalin-fixed paraffin-embedded section. The finding of 1 (0.6%) of the 162 interphase cells examined from the assays derived from formalin-fixed paraffin-embedded section with a split signal for BCL3 is below the 1–3% range for false positive signals for break-apart DNA probes.

## Discussion

We describe a case of a 26-year-old pregnant woman with a Xp11.2/TFE3 translocation RCC with a novel translocation involving chromosome 19, and a favorable clinical course. The tumor shows a combination of clear and eosinophilic granular voluminous cells in a mixed papillary and nested pattern with the presence of prominent psammomatous calcification. The diagnosis is confirmed immunohistochemically by strong nuclear immunoreactivity for TFE3, and cytogenetically by the presence of a Xp11.2 translocation. Translocations involving TFE3 induce overexpression of this protein, and can be specifically identified by immunohistochemistry [7]. Nuclear labeling for TFE3 is highly sensitive (97.5%) and specific (99.6%) for neoplasms bearing *TFE3 *gene fusions [7]. Argani et al. [7] reported that immunostaining of TFE3 is nuclear and should, in case of positivity, be obvious at low-power magnification. A reliable interpretation requires the absence of cytoplasmic labeling of tumor cells and absence of nuclear labeling in adjacent normal kidney. Diagnosis of Xp11.2 translocation RCCs, which remains underestimated in the absence of cytogenetic studies on fresh or frozen materials, is therefore now possible on archival paraffin blocks [7,17]. The histologic features of papillary and nested architecture, voluminous clear and eosinophilic cytoplasm, and numerous psammoma bodies of the case herein presented raised the possibility of RCC associated with a Xp11.2 translocation. However, the histology in some areas of the tumor and immunophenotype also suggested an oncocytic renal epithelial neoplasm with admixed clear/chromophobe cells and distal tubular differentiation (eosinophilic variant of chromophobe RCC versus appearance of RCC in patients with Birt-Hogg-Dube syndrome versus oncocytoma versus unclassified RCC). This emphasizes that Xp11.2 translocation RCCs can have a variety of gross and histologic appearances and significant heterogeneity within the tumor, and may have overlapping immunophenotype with other tumors. Hence, emphasizing the importance of performing cytogenetic analysis and including the TFE3 immunostain in panels for RCCs with unusual features, and probably for all RCCs in young patients.

Xp11.2 translocation RCC results from gene fusions between the TFE3 transcription factor gene located on chromosome Xp11.2 and one of six different gene fusion partners previously reported to date [1-24]. The molecular identity of five of these six gene fusion partners of TFE3 are known, whilst the identity of the sixth which is situated on chromosome 3 is not yet known [1-24]. The five known gene fusion partners of TFE3 are renal cell carcinoma papillary 1 (PRCC), alveolar soft part sarcoma locus (ASPL), polypyrimidine tract-binding protein-associated splicing factor (PSF), non-POU domain-containing octamer-binding (NonO, p54^nrb^), and clathrin heavy-chain (CLTC) genes, situated on chromosome loci 1q21, 17q25, 1p34, Xq12, and 17q23, respectively (Table 2) [1-24]. The t(X;17)(p11.2;q25) or TFE3-ASPL translocation RCC and alveolar soft part sarcoma (ASPS) contain the identical TFE3-ASPL fusion transcript; however, the t(X;17) translocation is consistently balanced (reciprocal) in the Xp11.2 translocation RCC and unbalanced in the ASPS [1]. The classical cytogenetic profile of the tumor herein reported indicates the majority of the tumor cells with a translocation involving chromosome region Xp11.2/TFE3 and chromosome region 19q13.1, suggesting that the tumor is a Xp11.2 translocation RCC with a novel translocation involving chromosome 19 and a unilocular cystic and mural multinodular gross appearance. Although, a cystic gross appearance is uncommon for Xp11.2 translocation RCCs, Suzigan et al. [16] recently reported a Xp11.2 translocation RCC in a 17-year-old female with multilocular cystic RCC-like features, who was alive with no evidence of disease four months after diagnosis. However, an RCC with translocation involving Xp11.2 to chromosome 19 has not been previously reported. Therefore, our study further expands the genetic spectrum of Xp11.2 translocation RCCs, in that we report a novel translocation, t(X;19)(p11.2;q13.1). Since this novel gene fusion partner of TFE3 at the 19q13.1 locus in the case herein presented is close to the *BCL3 *(19q13.3) gene located on the chromosome 19, interphase FISH analysis was performed on cultured and uncultured tumor cells using a dual-color break-apart DNA probe within the *BCL3 *gene on 19q13.3. The tumor cells were negative for the *BCL3 *gene rearrangement, indicating that the *BCL3 *gene is not the novel gene fusion partner of TFE3 in the case herein presented. Hence at this time, the molecular identity of the gene fusion partner of TFE3 at the 19q13.1 locus in the case herein presented remains unknown, since reverse transcriptase polymerase chain reaction (RT-PCR) analysis had yet to be successful at detecting and identifying the TFE3 fusion transcript. Interestingly, the neoplasm herein presented demonstrated some of the typical histological features observed in the TFE3-ASPL translocation RCCs, which harbor a t(X;17)(p11.2;q25) chromosome translocation, in that its cells had voluminous clear and eosinophilic cytoplasm and were associated with abundant psammoma bodies [5,14,17]. The case herein presented highlights the point that the morphology of Xp11.2 translocation RCCs harboring different gene fusions may overlap significantly. At the same time, the entire histologic architecture and grossly cystic morphology of the case herein presented are not classical of Xp11.2 translocation RCCs; and this might reflect the morphologic heterogeneity in translocation tumors, the influence of the novel translocation gene partner, both, or some other factor. Nonetheless, in many cases, the morphology may provide a clue to the specific gene fusion.

**Table 2:** Reported cytogenetic translocations involving Xp11.2/transcription factor E3 (*TFE3*)

Chromosome Translocation	Gene Fusion	Neoplasm	Source, Year
der(17)t(X;17)(p11.2;q25)	*ASPL-TFE3*	ASPS	Argani et al, 2001 [1]; Argani et al, 2007 [17]

t(X;17)(p11.2;q25)	*ASPL-TFE3*	RCC	Argani et al, 2001 [1]; Argani et al, 2007 [17]

t(X;1)(p11.2;q21)	*PRCC-TFE3*	RCC	Argani et al, 2007 [17]

t(X;1)(p11.2;p34)	*PSF-TFE3*	RCC	Argani et al, 2007 [17]

inv(X)(p11.2;q12)	*NONO-TFE3*	RCC	Argani et al, 2007 [17]

t(X;17)(p11.2;q23)	*CLTC-TFE3*	RCC	Argani et al, 2003 [8]; Argani et al, 2007 [17]

t(X;3)(p11.2;q23)	Unknown	RCC	Argani et al, 2007 [17]

*ASPL*, alveolar soft part sarcoma locus; ASPS, alveolar soft part sarcoma; *CLTC*, clathrin heavy chain; *NONO*, non-POU domain-containing octamer-binding; *PRCC*, papillary renal cell carcinoma; PSF, polypyrimidine tract binding protein-associated splicing factor; RCC, renal cell carcinoma

Macroscopically, Xp11.2 translocation RCCs may mimic conventional clear cell RCCs as they are usually tan-yellow, necrotic, and hemorrhagic. The most consistent histologic appearance of Xp11.2 translocation RCC is a carcinoma with mixed papillary and nested/alveolar architecture, composed of cells with clear and/or eosinophilic granular voluminous cytoplasm, discrete borders, vesicular chromatin, prominent nucleoli, and psammoma bodies [1-24]. However, the TFE3-PRCC variant is generally composed of cells of intermediate size and shows few psammoma bodies; this is in contrast to the TFE3-ASPL variant which generally shows large voluminous cells and presence of numerous psammoma bodies [1-24], features reminiscent of the case herein presented. In the case herein presented, the histological sections of the resected renal tumour showed numerous psammoma bodies, a feature that is rarely observed in conventional clear cell RCC but can be seen in papillary RCC [5,14,17]. The absence or paucity of true papillary groups with smooth-bordered contours, foamy macrophages, nuclear grooves, stromal inflammatory cells and a necrotic background did not support the diagnosis of papillary or conventional clear cell RCC [5,14,17]. Xp11.2 translocation RCC can occur in adults and may be aggressive cancers [9-24], and hence require morphologic distinction from conventional clear cell and papillary RCCs. Although they may be uncommon on a percentage basis, given the much higher prevalence of RCCs in adults compared with children, adult Xp11.2 translocation RCC may well outnumber their pediatric counterparts. Accurate histopathologic diagnosis, supported by confirmatory TFE3 immunohistochemistry (IHC), should allow this subset of adult RCCs to be delineated such that their clinicopathologic features can be analyzed further. On routine hematoxylin and eosin sections, Xp11.2 translocation RCC may overlap significantly with conventional clear cell and papillary RCCs in adults. The formation of papillae is, at most, rare in clear cell RCC, although pseudopapillary areas arising from degeneration of acinar structures may be difficult to distinguish from true papillae. Furthermore, clear cell RCC seldom, if ever, forms psammoma bodies, so, when present, psammomatous calcifications are evidence in favor an Xp11.2 translocation RCC [5,14,17]. IHC with conventional antibodies can be suggestive; whereas clear cell RCC express epithelial markers (cytokeratins and EMA) diffusely, Xp11.2 translocation RCCs are frequently negative or only weakly positive [5,14,17,19]. Although clear cells are uncommon in papillary RCC, clear cells may be seen in the areas of degeneration associated with hemosiderin deposition. Additionally, Xp11.2 translocation RCCs with prominent eosinophilic cytoplasm might be confused with type 2 papillary RCCs [5,14,17].

The most distinctive IHC feature of Xp11.2 translocation RCCs, absent in conventional clear cell and papillary RCCs, is a detectable nuclear staining for the chimeric (mutant) TFE3 protein [1-24], as in the case herein presented. The antibody used recognizes the C-terminal portion of the TFE3 protein, which is retained in all TFE3 fusion proteins. Because native TFE3 is known to be expressed constitutively and ubiquitously but not detectable in normal tissues by IHC, it is anticipated that all the different Xp11.2/TFE3 gene fusions consistently lead to the over-expression of TFE3 protein [5,7]. Previous studies have reported specific immunohistochemical patterns that are suggestive of the diagnosis of Xp11.2 translocation RCC, in the absence of TFE3 immunohistochemistry [5,14,17,19]. Generally, the expression of cytokeratins (AE1-AE3, Cam5.2, CK7, and EMA) and melanocytic markers (HMB45 and melan A) were rare and weak, the expression of vimentin was variable and weak, and that of E-cadherin, α-methylacyl-coenzyme A racemase, CD10 and RCC were common and strong in Xp11.2 translocation RCCs [5,14,17,19]. This typical immunoprofile of previously reported Xp11.2 translocation RCCs is consistent with that of the case herein presented, except for the strong EMA positivity and weak RCC positivity in our case. Generally, the absence of CK7 and EMA expression (CK7-, EMA-), together with the overexpression of E-cadherin and CD10 (E-cadherin+, CD10+) in Xp11.2 translocation RCCs have been suggested as useful tools in the differential diagnosis of conventional clear cell RCCs (CK7-, EMA+, E-cadherin-, CD10+) and papillary RCCs (CK7+, E-cadherin+) [5,14,17,19]. Hence, the typical immunoprofile of Xp11.2 translocation RCCs (CK7-, EMA-, E-cadherin+, CD10+) may be helpful when TFE3 immunostaining is not available or doubtful [5,14,17,19].

Xp11.2 translocation RCCs occur primarily, but not exclusively, in children and young adults, and is believed to be rather indolent even when diagnosed at advanced stages [1-8]. However, there have been increasing recent reports of Xp11.2 translocation RCC with aggressive clinical course in patients aged 16 or older (Table 3) [9-24]. These recent reports emphasize that, although the tumor morphology in adult Xp11.2 translocation RCC was similar to that in children, most of these adult patients had an aggressive clinical course [9-24]. Although these recent reports [9-24] indicate that Xp11.2 translocation RCCs may be inherently more aggressive in adults than in children; however, the relatively short follow-up periods currently available and the potential bias inherent on non-consecutive case series and case reports preclude a definitive prediction of behavior for individual patients. In contrast, the herein presented case of adult Xp11.2 translocation RCC occurring during the early second trimester of pregnancy had a favorable clinical course with the patient alive with no evidence of disease 27 months after diagnosis and surgery. Therefore, there seems to be clinicopathologic heterogeneity even in adults, but the clinical and/or molecular basis for this heterogeneity remains to be elucidated.

**Table 3:** Treatment, Follow-Up, and Prognosis of Recently Reported Xp11.2 Translocation Renal Cell Carcinomas in Patients Aged 16 or Older

			**Treatment**		**Pattern of Progression**			
					
Source, Year	Pts, No.	Age, y/Sex	Primary Treatment	Additional Treatment	Local Recurrence	Metastases	Follow-Up Time, y	Outcome
Dal Cin et al, 1998 [10]	1	53/F	RN	Resection of metastases	None	Lung, bone, liver, adrenal, retroperitoneal LNs	31	NS

Salles and Soto, 2005 [11]	1	58/F	RN	None	None	Renal hilar LNs	0.5	FOD

Mansouri et al, 2006 [12]	1	16/F	RN	Resection of metastases, ChemoRx, IFN-α, IL-2	Yes	Lung, bone, adrenal, lumbar-aortic LNs	NS	NS

Schinstine et al, 2006 [13]	1	57/F	RN	Resection of metastases	None	Lung	1	NS

Meyer et al, 2007 [14]	5	32.6 (mean)/5M	4 RN; 1 renal Bx	Resection of metastases, ChemoRx, XRT, IL-2, investigational agents	Yes	Lung, bone, liver, brain, mediastinal LNs	1.5 (mean)	2 DOD

Rais-Bahrami et al, 2007 [15]	1	23/M	RN	Resection of metastases, ChemoRx, investigational agents	None	Lung, liver, retroperitoneal, supraclavicular, cervical and mediastinal LNs	17	DOD

Suzigan et al, 2007 [16]	1	17/F	PN	None	None	None	0.33	FOD

Argani et al, 2007 [17]	28	22–68 (range)/22F, 6M	NS	NS	NS	5 hematogenous metastases	NS	2 DOD

Armah and Parwani, 2008 [18]	1	33/M	RN	Resection of metastases, ChemoRx, XRT, IFN-α, IL-2	None	Lung, liver, bone, brain, mediastinal LNs	0.58	DOD

Camparo et al, 2008 [19]	31	24.6 (mean)/18F, 13M	30 RN; 1 renal Bx	Resection of metastases, ChemoRx, XRT, IFN-α, IL-2, investigational agents	NS	Lung, liver, bone, LNs	0.5–7.67 (range)	5 DOD

Wu et al, 2008 [9]	3	17–20 (range)/1F, 2M	3RN	Resection of metastases, investigational agents	Yes	Retroperitoneal LNs	1.7–3.4 (range)	1 DOD

Gellar et al, 2008 [20]	4	16–17 (range)/3F, 1M	4RN	ChemoRx, IFN-α, IL-2, investigational agents	None	Lung, liver	1.17–15.42 (range)	1 DOD

Hintzy et al, 2008 [21]	6	28–42 (range)/4F, 2M	NS	NS	NS	3 hematogenous metastases	2.67 (mean)	1 DOD

Komai et al, 2009 [22]	6	24–59 (range)/2F, 4M	6RN	Resection of metastases, IFN-α, IL-2	None	Lung, liver, LNs	0.75–11 (range)	2 DOD

Bovio et al, 2009 [23]	1	20/F	RN	NS	None	Placenta, bone, retroperitoneal LNs	2	NS

Koie et al, 2009 [24]	1	28/M	RN	Resection of metastases, IFN-α, IL-2	Yes	Lung, liver, adrenal, spleen, pancreas, psoas muscle, mesentery, descending colon, retroperitoneal LNs	2	DOD

Bx, biopsy; ChemoRx, chemotherapy; DOD, died of disease; F, female; FOD, free of disease; IFN-α, interferon-α; IL-2, interleukin-2; LNs, lymph nodes; M, male; No., number; NS, not specified; PN, partial nephrectomy; Pts, patients; RN, radical nephrectomy; XRT, radiotherapy; y, years

Renal tumors are rare in pregnancy and occur in approximately 1 per 1,000 pregnancies [28]. RCC is the most common renal tumor reported in pregnancy, accounting for about half of all primary renal tumors during pregnancy [28]. Over 80 cases of RCC occurring during pregnancy has been reported in the English Language medical literature [28]. Most cases are incidentally diagnosed during pregnancy (as in the case herein presented), and usually present as a large palpable mass, probably because of more frequent abdominal and ultrasonographic examinations during pregnancy [28]. The most common histologic subtypes of RCC that have been reported during pregnancy include conventional clear cell RCC, papillary RCC and chromophobe RCC. To the best of our knowledge, the case herein presented is the first report of Xp11.2 translocation RCC occurring during pregnancy. Bovio et al. [23] recently reported the case of a 20-year-old pregnant female with placental, bone and retroperitoneal lymph node metastases two years after radical nephrectomy for Xp11.2 translocation RCC, but no evidence of fetal metastasis. Hence, the initial diagnosis of Xp11.2 translocation RCC occurred prior to her pregnancy. Bovio et al. [23] stated that given the need for systemic therapy and risk of harm to the fetus, a Cesarean section was performed at 33 weeks gestation. However, they did not specify whether subsequent systemic therapy was given, what specific type of systemic therapy was given (if any), the fate of the bone and retroperitoneal lymph node metastases, and the final patient outcome (whether she was alive with no evidence of disease, was alive with progressive disease, or died of disease). Surgical resection (partial or radical nephrectomy) is the preferred therapy in patients with lower stage (potentially curable) tumors, and may be performed in pregnant women. Nevertheless, one of the major issues in the management of cancer in pregnancy is timing of surgery.

The recommendation of surgery during the first and third trimester of pregnancy is universally accepted, but the recommendation of surgery during the second trimester is controversial [28]. It should be considered that delaying surgery can potentially be harmful for the risk of metastasis. However, when proposing surgery during the second trimester of pregnancy, one should be aware of the potential risks of surgical manipulations, including uterine contractions leading to fetal distress or even spontaneous abortion. In the case herein presented, immediate surgery was probably the best treatment option for RCC occurring during early second trimester of pregnancy. Because placental and fetal metastases are rare, specific surveillance guidelines and prognostic information are unclear for the infant who does not present with metastases at birth, as in the case herein presented.

While the increasing numbers of case reports of RCC arising during pregnancy could represent a chance occurrence, there is some evidence that there may be an increased risk of the development of some neoplasms (including RCC) during pregnancy, possibly related to hormonal changes (including increased estrogen and beta β-hCG levels) and increased immune tolerance [29]. Additionally, the increasing rates of RCC in women compared to men over the past 30 years, the presence of steroid hormone receptors in RCC tumor cells, the induction of renal tumors in experimental animals with diethylstilbestrol and estradiol, and the observation that obesity is a consistent risk factor for RCC suggest a possible role of reproductive or hormonal factors in RCC [29]. Kabat et al. [29] recently demonstrated that high parity may be associated with increased risk of RCC, and that oral contraceptive use may be associated with reduced risk. Indeed, compared with nulliparous women, parous women were at increased risk [hazard ratio (HR) = 1.78, 95% confidence interval (CI) = 1.02–3.09] of RCC, and there was a significant gradient of risk with increasing levels of parity: relative to nulliparous women, women who had ≥ 5 pregnancies lasting 4 months or more had a 2.4-fold risk (HR = 2.41, 95% CI = 1.27–4.59, P for trend 0.01) [29]. Ever use of oral contraceptives was associated with a modest reduction in risk. No associations were observed for age at first live birth or use of hormone replacement therapy [29].

## Conclusion

The patient herein presented was older than typically described for Xp11.2 translocation RCC. Although tumor morphology showed some similar features to those in the other recently reported cases of adult Xp11.2 translocation RCC with aggressive clinical courses [9-24], our patient had a more favorable clinical course, a cystic morphology, and a novel translocation involving chromosome 19. Hence, the consistent immunohistochemical staining for TFE3 in all RCC with unusual gross and/or histologic features, regardless of patient age, is likely to expand the spectrum of Xp11.2 translocation RCC with respect to age, clinical behavior, morphology, and molecular abnormalities.

## Consent

Consent was received from the patient before publication.

## Competing interests

The authors declare that they have no competing interests.

## Authors' contributions

HBA participated in the histopathological evaluation, performed the literature review, acquired photomicrographs and drafted the manuscript. US performed the cytogenetic and molecular (FISH) analyses, and critically revised the manuscript for important intellectual content. AVP and SIB conceived and designed the study, reviewed the histopathological diagnosis and revised the manuscript for important intellectual content. All authors read and approved the final manuscript.
